# A Brief Review of Analytical Methods for the Estimation of Allopurinol in Pharmaceutical Formulation and Biological Matrices

**DOI:** 10.1155/2021/5558651

**Published:** 2021-06-05

**Authors:** Hemraj Sharma, Hari Prasad Sapkota, Nim Bahadur Dangi

**Affiliations:** ^1^Department of Pharmacy, Shree Medical and Technical College, Bharatpur, Chitwan, Nepal; ^2^Pharmaceutical Sciences Program, School of Health and Allied Sciences, Faculty of Health Sciences, Pokhara University, Kaski, Nepal

## Abstract

This review article represents the collection and discussion of various analytical methods available in the literature for the determination of allopurinol (ALLP) in pharmaceutical and biological samples consisting of HPLC, UV-visible method, near-IR spectroscopy, spectrofluorometry, capillary electrophoresis, polarography, voltammetry, and hyphenated techniques such as LC-MS, LC-MS/MS, UPLC-MS/MS, and GC-MS. The anticipated review provides details about the comparative utilization of various analytical techniques for the determination of ALLP. The present review article can be effectively explored to conduct future analytical investigation for the estimation of ALLP.

## 1. Introduction

Hyperuricemia is a condition characterized by abnormally elevated levels of serum urate in which there is major increase in purine metabolism subsequent to the fast lysis of malignant cells occurring in patients with large tumor burden, both spontaneously and after aggressive chemotherapy [1]. It results from the impairment with uric acid renal clearance, among patients with kidney diseases or iatrogenic adverse events. This metabolic complication may be potentially dangerous since uric acid, via precipitating in renal tubules, can cause acute renal failure [2]. The drugs used for hyperuricemia are used with the intent of lowering the quantity of uric acid in blood, and this may be obtained by reducing the formation of uric acid or by increasing the clearance of formed uric acid. ALLP has structural similarity with hypoxanthine (natural purine base) and acts by inhibiting the production of uric acid [3].

## 2. Experimental

### 2.1. Physiochemical Properties

**Figure 1:**
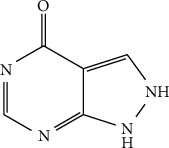
Structure of allopurinol.

ALLP is chemically 1H-pyrazolo[3,4-d]pyrimidin-4-ol (Figure 1). It is a tautomeric mixture of 1H-pyrazolo[3,4-d]pyrimidin-4-ol and 1,5-dihydro-4H-pyrazolo[3,4-d]pyrimidin-4-one [4]. The molecular weight and molecular formula of ALLP are 136.11 g mol^−1^ and C_5_H_4_N_4_O, respectively. It is sparingly soluble in water and in ethanol (95%) and practically insoluble in chloroform and in ether. It is soluble in dilute alkali hydroxides solutions. It is white to almost white, crystalline powder [5].

### 2.2. Mechanism of Action

ALLP interferes with the catabolism of purines by interfering with xanthine oxidase enzyme and inhibits its effect which is responsible for the interconversion of hypoxanthine to xanthine and too uric acid. Interestingly, ALLP is not only a blocker, but also a substrate of xanthine oxidase, and the deriving metabolite, oxypurinol (OXP), is a potent inhibitor of xanthine oxidase itself and may be considered responsible for much of its pharmacological effect [6]. The reduction in the production of uric acid lowers its plasma and urinary levels and favors the formation of hypoxanthine and xanthine, which can be considered as its precursors [7]. Hypoxanthine is more soluble in water, whereas xanthine is yet less soluble compared to uric acid [8]; hence, the use of ALLP may potentially amplify the threat of precipitating xanthine in renal tubules [9].

### 2.3. Pharmacokinetics and Pharmacodynamics

ALLP can be administered either orally or intravenously. The oral bioavailability is about 67 to 90% with a peak plasma concentration occurring within one hour; the volume of distribution is approximately 1.6 L kg^−1^ [10, 11]. It is principally metabolized by aldehyde oxidase to the active compound OXP. The peak plasma concentration of OXP occurs within 3–5 hours. Mean elimination plasma half-life ranges between 0.7 and 1.5 hours for ALLP and 18–40 hours for OXP [12]. ALLP is excreted in urine (less than 10% unchanged, 70% as OXP) and in feces (20%).

The main objective of the present work is to describe various simple and sophisticated analytical methods for determining ALLP in various formulations and matrices. The compiled data may be explored for the studies on analysis of ALLP. The major analytical methods include UV-visible spectrophotometric method, IR spectroscopy, spectrofluorimetric method, chromatographic methods like high-performance liquid chromatography (HPLC) and gas chromatography (GC), hyphenated techniques like gas chromatography-mass spectrometry (GC-MS) and liquid chromatography-mass spectrometry (LC-MS), and miscellaneous methods.

### 2.4. Pharmaceutical Methods of ALLP

#### 2.4.1. Pharmacopeial Methods

ALLP is an official drug in the Indian Pharmacopoeia (IP), British Pharmacopoeia (BP), and United States Pharmacopeia (USP). IP and BP have reported an HPLC procedure for the analysis of ALLP using UV-visible spectrophotometer by measuring the absorbance of drug at 250 nm. The pharmacopeia has used 563 as the specific absorbance of ALLP at 250 nm. The drug was extracted with sodium hydroxide, followed by HCl, and HCl was used as a blank [13, 14]. USP describes the analysis of ALLP using chromatographic method. HPLC analysis was done using reversed-phase column (30 cm × 4 mm), using mobile phase composed of 0.05 M solution of monobasic ammonium phosphate at a flow rate of 1.5 mL min^−1^, and detection of wavelength was made at 254 nm [15].

## 3. Analytical Methods

### 3.1. UV-Visible Spectrophotometric and Spectrofluorimetric Methods (UV-Vis Methods)

**Figure 2:**
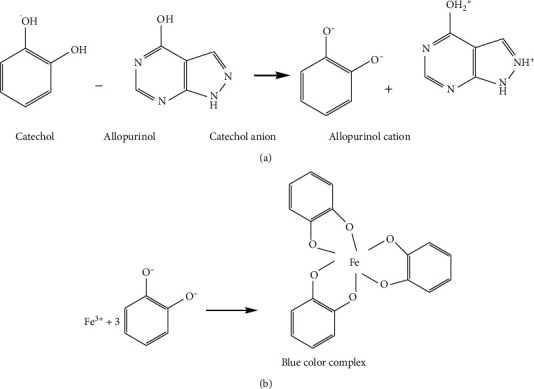
(a) Ionization of catechol along with ALLP and (b) blue color complex formation.

Up till now, a number of UV spectrophotometric methods have been reported for determining ALLP. Khayoon has developed rapid, cheap, reliable, and simple spectrophotometric method for the quantitative analysis of ALLP in tablet formulation based on the reaction of ALLP with catechol reagent and Fe(II) to form a blue soluble complex (Figure 2) which was measured at *λ*_max_ 580 nm.

Optimization of the developed method was studied on various parameters like the order of addition of reagent, volume of ferrous solution, catechol solution, and time of stability. The results stated that adding catechol followed by ALLP and ferrous solution gave good result. 2 ml of ferrous solution and 3 ml of catechol gave high absorbance whereas the authors found that the color complex was stable up to 2 hours in stability study. The drug was linear over the concentration range 2–10 *μ*g mL^–1^ with a good recovery of 100% and RSD of 1.0%–1.3% [16].

Patel et al. established spectroscopic method for simultaneous estimation of ALLP and *α*-lipoic acid (LA) in combined tablet dosage forms using AUC and absorbance correction method. ALLP is estimated by absorption correction method at 250 nm whereas *α*-lipoic acid was determined by AUC area at 310 nm to 390 nm. The drugs seem to be linear at the concentration range of 10–50 *μ*g mL^–1^ with a mean recovery of 101.35% and 101.41% for ALLP and LA, respectively. The limit of detection (LOD) was 0.16 *μ*g mL^–1^ and 2.55 *μ*g mL^–1^ for ALLP and *α*-lipoic acid, respectively. The limit of quantification (LOQ) was 0.5 *μ*g mL^–1^ and 7.74 *μ*g mL^–1^ for ALLP and *α*-lipoic acid, respectively. Precision of the developed method was evaluated as per interday and intraday and was found to be <2% [17].

**Figure 3:**
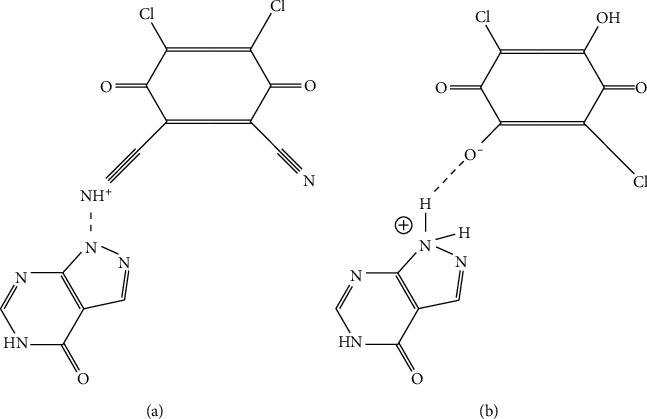
(a) Structure of the ALLP-DDQ CT-complex; (b) structure of the ALLP-p-CLA CT-complex.

Refat et al. have established a spectrophotometric micro determination of ALLP via charge-transfer formation by using 2,3-dichloro-5,6-dicyano-p-benzoquinone (DDQ) and 3,6-dichloro-2,5-dihydroxy-p-benzoquinone (p-CLA) reagents as shown in Figure 3. The absorbance was measured at *λ*_max_ of 450 and 515 nm for ALLP-DDQ and ALLP-p-CLA CT complexes, respectively, against reagent blanks.

The drug obeyed Beer's limit over the concentration of 2.50–60.00 *μ*g mL^–1^ for DDQ and 5.00–50.00 *μ*g mL^–1^ for p-CLA method, respectively, with a mean recovery of 98.40–100.7% and 98.20–100.4% for ALLP-DDQ and ALLP-p-CLA CT complexes, respectively. Precision of the developed method was evaluated as per interday and intraday and was found to be <2% (0.12–0.94%). The LOD was 7.96 *μ*g mL^–1^ and 1.70 *μ*g mL^–1^ for ALLP-DDQ and ALLP-p-CLA CT complexes, respectively. LOQ was 26.53 *μ*g mL^–1^ and 5.68 *μ*g mL^–1^ for ALLP-DDQ and ALLP-p-CLA CT complexes, respectively [18].

Abdel-Hay et al. have derived a derivative spectrophotometry for the in vitro determination of ALLP and uric acid (UA) mixtures in urine. UA was determined by measuring the second derivative (2D) value at 293 nm in 0.1 N hydrochloric acid, while ALLP was determined by the first derivative (1D) value at 284 nm in 0.1 N sodium hydroxide. The drug obeyed Beer's limit over the concentration of 0.2–1.2 mg dL^−1^ for both drugs with a mean recovery of 100.14% and 100.23 for ALLP and uric acid, respectively. The interday precision was evaluated through replicate analysis of urine samples spiked with ALLP at different concentration levels, and the % RSD was 1.39, indicating the high precision of the method [19].

Shoukrallah et al. published a research article, stating the quantitative analysis of ALLP and flucytosine (FC), in commercial tablets using differential UV spectrophotometric method. Because the active ingredients (ALLP and FC) exhibit different UV spectra according to the pH of the solutions to be analyzed, it was favorable to use the differential spectroscopy. Both drugs showed linear relationships obeying Beer's law over a concentration range of 0.25–3.5 *μ*g mL^−l^. The mean recoveries were found to be 99.75 and 99.84% for ALLP and FC, respectively. The coefficients of variation worked out to 0.80 and 0.94% for AP and FC, respectively [20].

Mohamed et al. have established simple and accurate spectrophotometric method that does not require many steps of mathematical equations or software for processing of the recommended data for ALLP and Lesinurad (LSD) in recently approved FDA pharmaceutical preparation. LSD was analyzed by zero-order spectrophotometric method at 290 nm, and ratio difference and ratio derivative spectrophotometric methods were applied for quantitative analysis of ALLP. For ratio difference method, the wavelengths 252 and 228 nm were plotted against the corresponding ALLP concentrations, and ratio derivative spectrophotometric method was analyzed by first-order method using Δ*λ* = 2 and scaling factor = 10 at 240 nm. The drugs obeyed Beer–lambert law over the concentration range 3–45 *μ*g mL^–1^ and 1–16 *μ*g mL^–1^ with a mean recovery of 100.27% and 99.56–99.68% for LSD and ALLP, respectively. The LOD was 0.201 *μ*g mL^–1^ and 0.203 *μ*g mL^–1^ for ALLP by ratio difference and ratio derivative method and 0.903 *μ*g mL^–1^ for LSD. The LOQ was 0.610 *μ*g mL^–1^ and 0.615 *μ*g mL^–1^ for ALLP by ratio difference and ratio derivative method and 2.736 *μ*g mL^–1^ for LSD. The repeatability and intermediate precision was evaluated through replicate analysis of ALLP at different concentration levels, and the % RSD was <2% (0.458–0.951%), indicating the high precision of the method [21].

Bedair et al. demonstrated a spectrofluorimetric method for determination of cimetidine (CM), thiabendazole (TB), carbimazole (CB), and ALLP on the basis of fluorescence quenching of mercurochrome (MER) in an aqueous alkaline medium and measuring the MER fluorescence (*λ*ex, = 365 nm and *λ*em = 535 nm) before (Fo) and after (F) the addition of quencher [22] using the corresponding buffer as blank and calculating ∆F(=Fo-F) and Fo/F ratio for the analysis. The mean recovery was found to be in the limit of 99.75–101.04% [23].

Attia et al. have established highly sensitive, selective, and accurate fluorescence spectroscopic methods for quantitative analysis of LSD and ALLP in pharmaceutical formulations and in human plasma. For LSD, *λ*ex was established at 288 nm and *λ*em at 343 nm, whereas ALLP was analyzed with *λ*ex of 465 nm and *λ*em of 535 nm. Calibration graphs were linear up to 0.25–4.0 *μ*g mL^–1^ for LSD and 0.2–20 *μ*g mL^–1^ for ALLP. The mean recovery for tablet formulation was found to be 99.55 ± 0.99% and 100.18 ± 1.84% for LSD and ALLP, respectively, and for spiked human plasma it was found to be 97.19 ± 0.85% and 95.79 ± 1.82% for LSD and ALLP, respectively. The LOD was 0.056 *μ*g mL^–1^ for ALLP and 0.069 *μ*g mL^–1^ for LSD, whereas LOQ was found to be 0.171 *μ*g mL^–1^ and 0.210 *μ*g mL^–1^ for ALLP and LSD, respectively. The repeatability and intermediate precision were evaluated through replicate analysis of ALLP at different concentration levels, and the % RSD was <2% (0.573–1.051%), indicating the high precision of the method. The authors also established the robustness of the developed method by using constancy of the fluorescence intensity with small variations in the optimum conditions such as pH (±0.2) and buffer volume (±0.2 mL) of LSD and ALLP in addition to 4-chloro-7-nitrobenzo-2-oxa-1,3-diazole (NBD-Cl) volume (±0.2 mL) and heating temperature (±10°C) for ALLP only. It was confirmed that there was no considerable effect on the fluorescence intensity of LSD and ALLP resulted from minor changes of procedure parameters [24]. All of the UV-visible and spectrofluorimetric methods of ALLP are summarized in Table 1.

### 3.2. High-Performance Liquid Chromatography Method

Several HPLC methods were reported for the analysis of ALLP in combination with other drugs. Dastiagiriamma has developed HPLC method of ALLP and Lesinurad (LSD) in the API and marketed formulation. Separation of ALLP was accomplished on a Zorbax C18 column by using methanol: phosphate buffer as the mobile phase at a flow rate of 1.0 ml/min. Further, UV (ultraviolet) detection of drug was measured at 255 nm. The application of the developed method in marketed formulation showed mean recoveries in the range of 99%–100.1% while LOD and LOQ were found to be 0.07 *μ*g mL^−1^ and 0.2 *μ*g mL^−1^, respectively [25].

Khader et al. have established HPLC method for ALLP and LSD in API and pharmaceutical formulation. Separation was carried out on a Inertsil ODS by using 0.1% trifluoroacetic acid and methanol as the mobile phase at a flow rate of 1.0 mL min^−1^. Further, UV (ultraviolet) detection of drug was measured at 255 nm. The application of the developed method in marketed formulation showed mean recoveries in the range of 100.48–100.96% for ALLP and 100.20–100.75% for LSD. LOD and LOQ were established by S/N method. LOD was found to be 3.03 and 2.98 and LOQ 10.02 and 9.98, respectively [26].

Reinders et al. have reported HPLC method for ALLP and oxypurinol (OXP) in human serum using reversed-phase LiChrospher 100 RP-18 column and 0.02 M sodium acetate as the mobile phase at a flow rate of 1.0 mL min^−1^, an injection volume of 40 *μ*L, and UV detection at 254 nm. The application of developed method was established on the serum of 66 patients which showed <0.5–4.3 mg L^−1^ for ALLP and <1.0–39.2 mg L^−1^ for OXP, respectively, for prescribed 300 mg/day dose of drug. LOD was found to be 0.1 mg L^−1^ (4 ng) and 0.2 mg L^−1^ (8 ng) for ALLP, and LLOQ was found to be 0.4 mg L^−1^ (16 ng) for ALLP and 0.6 mg L^−1^ (24 ng) for OXP [27].

Rajkumar et al. have established HPLC method for simultaneous quantitative estimation of ALLP and alpha-lipoic acid (LPA) in tablets, using Enable C18 G (250 × 4.6 mm; 5*μ*) as a stationary phase (50 : 50 v/v) and acetonitrile: 0.02 M ammonium acetate buffer (pH adjusted to 4.6) as a mobile phase with flow rate of 0.8 mL min^−1^, and the detection was done at 210 nm using a UV detector. Percentage mean recoveries were in the range of 98–102%, and LOD was found to be 3 ng mL^−1^ for ALLP and 0.5 *μ*g mL^−1^ for LPA, while LOQ was obtained as 10 ng mL^−1^ for ALLP and 1 *μ*g mL^−1^ for LPA [28].

Fadul has developed an UV and HPLC method for determination of ALLP in tablet dosage form using C8 column and 70% buffer solution (monobasic ammonium phosphate 0.05 M with 30% acetonitrile and methanol 1 : 1) as the mobile phase at a flow rate of 1.0 mL min^−1^. The drug was analyzed at 250 nm. Analytical method development results indicated good regression value with assay value of 98.43%. The LOD and LOQ were found to be 0.051 *μ*g mL^−1^ and 0.156 *μ*g mL^−1^, respectively [29].

dos Santos et al. have developed stability-indicating assay method for the simultaneous determination of ALLP and ketoconazole (KTZ) in the pharmaceutical form of capsules, the combination of which was used for the treatment of canine leishmaniasis. InertSustain® C18 column (4.6 × 100 mm × 3 *μ*m) was used as stationary phase and acetonitrile: water (52 : 48 v/v) with pH adjusted to 3.0 as a mobile phase, with a flow rate of 0.45 mL min^−1^. ALLP was detected at 250 nm KTZ at 225 nm. The LOD obtained was 0.0858 and 0.2599 *μ*g mL^−1^ for ALLP and KTZ, respectively. The LOQ was 0.0331 and 0.1004 *μ*g mL^−1^ for ALLP and KTZ, respectively. Additionally, robustness was indicated by the Plackett–Burman model, and the method was not significantly influenced by any of the variations [30].

Palmisano et al. have developed HPLC method with polarographic and voltammetric anodic detection: simultaneous determination of ALLP, OXP, and uric acid (UA) in body fluids by using a reversed-phase column (PerkinElmer RP-8, 10 pm, 250 × 4.6 mm) as a stationary phase and 0.025 M phosphate buffer pH 6.1 with 6–8% of methanol as a mobile phase, at a flow rate of 1.5 mL min^−1^. The current-concentration plots of samples were straight-lined over the range 2–2000 ng using the oxidative mode, with lowest detectability of 0.2 *μ*g mL^−1^. Patients taking 300 mg/day ALLP were analyzed by voltametry and polarographic method. Glassy carbon wall jet detector operating at +1.2 V vs. SCE was used for voltammetry analysis and oxidative mode with applied potential +0.24 V vs. Ag/AgCl as reference electrode, and dropping mercury electrode was used for polarography analysis. Uric acid showed good resolution when the operating potential was dropped from +1.2 V to +0.7 V by voltammetry method [31].

Brown and Bye have developed HPLC method with ion exchange chromatographic detection of ALLP and OXP in biological samples like human plasma and urine. This method is based on high-performance ion-exchange chromatography with proficient sample purification using resin (Chelex-100) in the cuprous form. ALLP has good linear calibration curves in the range of 0.068–1.36 *μ*g mL^−1^ in plasma and 0.68–136 *μ*g mL^−1^ in urine, and OXP has 0.076–15.2 *μ*g mL^−1^ and 15.2–304 *μ*g mL^−1^ in plasma and urine, respectively. The analysis was carried out by using HPLC precolumn and columns with configuration of stainless steel tubes 6.35 mm (1/4 in.) OD, 4.5 mm ID, 30 mm and 70 mm long, respectively, packed with Aminex A-27 (12–15 *μ*m) anion-exchange resin. The analysis was carried out at 254 nm with isocratically using ammonium acetate pH 8.7 at a flow rate of 1 mL min^−1^. The use of ligand exchange resins shortens the retention time to 25–30 min by isocratic elution when compared to gradient elution [32].

Tada et al. have developed HPLC method with UV detection of ALLP and OXP in human serum in the presence of sulfanilamide as internal standard. Separation was achieved by using C18 (particle size 10 *μ*m, Waters Co., Milford, MA, USA) with RCM 8 × 10 (Waters compressing modulator) connected to a precolumn (3.9 × 20 mm i.d., Waters guard column) and packed with Resolve C18 which was used for HPLC. The mobile phase was 2% (v/v) acetonitrile solution containing 100 mm potassium phosphate solution (pH 4·0) and 0.5 mm tetra-n-butylammonium hydrogen sulphate. The analysis was carried out at 260 nm, and the mobile phase was pumped at a flow rate of 2.0 mL min^−1^. The recovery of this method was obtained (97.4–101% for ALLP and 93.2–98.1% for OXP, respectively). The % RSD of this method was <5.1% for ALLP and <5.6% for OXP intraday. The % RSD of interday was <6·6% for ALLP over the range of 0.5–5·0 *μ*g mL^−1^ and <5·2% for OXP over the range of 0.4–20 *μ*g mL^−1^. The LOQ was 6 ng for ALLP and 4.8 ng for OXP. To evaluate potential interference by compounds derived from xanthines, samples were spiked with uric acid, hypoxanthine, xanthine, theophylline, theobromine, 1,7-dimethylxanthine, and caffeine, and the result revealed that there were no interfering peaks at retention times corresponding to those of ALLP and OXP [33].

Putterman et al. have developed HPLC and GC method for simultaneous analysis of hypoxanthine (HPX), xanthine (XN), ALLP, OXP, and UA in standard mixtures and physiological fluids. For HPLC, separation was carried out isocratically with an Altex Model 310 (Altex Instruments, Berkeley, Calif.) high-pressure liquid chromatograph equipped with a dual wavelength (254 and 280 nm) detector with mobile phase of acetonitrile-buffer (1 : 1). For GC, separation was carried on SE-30 (4% on 100/120 mesh SUPELCOPORT) glass column, 0.2 × 180 cm, with flow of nitrogen as carrier gas at 20 mL min^−1^. The injection temperature was 200°C, and the flame ionization detector temperature was maintained at 300°C. HPLC requires no prior derivatization, uses isocratic elution with a buffer containing no organic solvent, and has 50- to 100-fold greater sensitivity than GC, whereas the interference effect was minimal in GC as some metabolite present in the urine sample interfered with HPLC. However, with isocratic elution employing a completely aqueous buffer at pH 4.5, the authors analyzed the compounds of interest without interference from compounds such as pseudouridine which is known to be present at high levels in the urine of cancer patients. With two sensitive techniques, GC and HPLC, the identities of all five compounds of interest were highly possible. Prepurification for HPLC was done by deproteinization, whereas for GC it was done by using Sephadex G-10 equilibrated with 0.156 M triethyl ammonium acetate, pH 5.0. The recoveries of the five compounds of interest were in the following ranges: UA, 89–108%; hypoxanthine, 95–110%; xanthine, 88–106%; OXP, 97–110%, and ALLP, 95–105%. The application of the developed method has been established by analyzing urine sample from cancer patients having ALLP by both methods. Excellent correlation was obtained for each of the compounds except ALLP in HPLC, but GC showed low but measurable levels of ALLP in these samples [34].

Boulieu et al. have developed an HPLC method for simultaneous analysis of HPX, XN, ALLP, OXP, and UA in physiological fluids. For HPLC, separation was carried out using ODS column (15 cm × 4.6 mm ID) and a precolumn (5 cm × 4.6 mm ID), used as a guard column. The mobile phase consisted of 0.02 M potassium dihydrogen phosphate, the pH of which was adjusted to 3.65 with orthophosphoric acid. The flow rate was 1.5 mL min^−1^, and the detection was carried out at 254 nm. Calibration curves were linear from 0.15 to 20 mg L^−1^ for ALLP and OXP and from 0.50 to 50 *μ*mol L^−1^ for HPX and XN. LOD was found to be 1.5 ng for ALLP and OXP, 2.5 pmol for HPX, and 5.0 pmol for XN. Precision in terms of reproducibility and accuracy were determined on spiked plasma. The interassay coefficient of variation for the analysis of ALLP and OXP over the concentration range 0.5–5 mg L^−1^ was found to be about 3% for both ALLP and OXP, for HPX and XN it was found to be 1.5%, and recovery was almost 100%. The authors observed that the HPX and XN levels in plasma and especially in urine from patients under ALLP therapy are higher than those obtained in their study of healthy subjects [35].

Hung et al. have developed HPLC method with UV detection of ALLP and OXP in human serum in the presence of sulfanilamide as internal standard. Separation was achieved by using 100 mm × 4.6 m ID and slurry packed with 5 *μ*m Hypersil ODS (Shandon, London, UK). The mobile phase was 20 mM disodium hydrogen phosphate dihydrate and was adjusted to pH 2.0 with orthophosphoric acid. The analysis was carried out at 254 nm, and the mobile phase was pumped at a flow rate of 2.0 mL min^−1^. Trichloroacetic acid and perchloric acid were found to be quite more effective in the removal of interfering substances than when either was used alone. The recovery of this method was obtained (97.4–101% for ALLP and 93.2–98.1% for OXP, respectively). The % RSD of this method was <8% for ALLP over the range of 0·05–5.0 *μ*g mL^−1^. The LOQ for ALLP and OXP was 30 ng mL^−1^ [36]. All of the HPLC/UPLC methods of ALLP are summarized in Table 2.

### 3.3. Gas Chromatography

Milleret al. have developed a gas chromatography method for the analysis of ALLP in phenanthrene tablet as an internal standard. ALLP was analyzed by derivatization technique, which involves silylation reaction consisting of stationary phases of 3% OV-101 and 3% OV-17 on Chromosorb W/HP AW-DMCS (100-120 mesh). The temperature of column was maintained at 150^0^C for analysis of ALLP. Qualitative analysis was carried out in terms of retention indices on both columns with OV-101 and OV-17 stationary phases. All the substances yielded single symmetrical elution peaks on both columns, for which ALLP was chromatographed at 1612.6 for OV-101 and 1723.6 for OV-17, respectively. The amount of ALLP in a tablet was found to be 97.44 ± 0.83 mg [37]. No validation parameters were analyzed in this method.

### 3.4. HPTLC-Densitometry Method

Pandya et al. have developed highly sensitive high-performance thin layer chromatography (HPTLC) method for the simultaneous determination of ALLP and OXP in human plasma and ALLP in tablet dosage form. Separation was achieved on aluminium plates precoated with silica gel 60G F254, using methanol: chloroform: ammonia (2.0 : 7.9 : 0.1, v/v/v) as the developing solvent system. Densitometric measurements were established at 206 nm, and the results of retention factors (Rf) were 0.38 ± 0.01 and 0.65 ± 0.01 for ALLP and OXP, respectively. The regression plots were linear (*r*^2^ > 0.9993) over the concentration range of 100–700 ng/band for both analytes. The LOD and LOQ of the method were 19.56 and 59.29 ng/band for ALLP and 19.01 and 57.59 ng/band for OXP, respectively, for plasma samples. For spiked plasma samples, protein precipitation with formic acid in acetonitrile afforded mean recovery of 84.67% and 86.21% for ALLP and OXP, respectively. The recovery of ALLP and OXP in tablet formulation was in the range of 99.68–101.72% [38].

### 3.5. Hyphenated Techniques

#### 3.5.1. LC-MS

Kasawar et al. have developed highly sensitive LC-MS/MS method for determination of ALLP and OXP in human plasma using lamivudine as an internal standard. Chromatography separation was carried out in Waters Symmetry Shield RP_8_, 150 mm × 3.9 mm, 5 *μ*m columns using a mixture of 0.01% formic acid in water and acetonitrile in the ratio of 95 : 05 (v/v) as the mobile phase. Negative electrospray was used for detecting and quantifying the analyte by mass spectrometry. The drug was linear up to 0.01–10 *μ*g mL^−1^ with a lower limit of quantification of 0.01 *μ*g mL^−1^ for both ALLP and OXP. In this research, dilution integrity test, hemolysis and anticoagulant effect, and matrix effect studies were reported [39].

Rathod et al. reported a pharmacokinetic/bioequivalence study based on LC-MS/MS method for the simultaneous determination of ALLP and its active metabolite, OXP in human plasma. The analytes were separated on Hypersil Gold (150 mm × 4.6 mm, 5 *μ*m) column using 0.1% formic acid-acetonitrile (98 : 2, v/v) as the mobile phase. Positive electrospray was used for the detection and quantification of the analyte by mass spectrometry. The proposed method obeyed good calibration curve in the range of 60.0 to 6000 ng mL^−1^ for ALLP and 80.0–8000 ng mL^−1^ for OXP. Moreover, the absence of matrix interference was confirmed by IS-normalized matrix factors and concentration of drugs from different plasma source [40].

Liu et al. also reported a pharmacokinetic/bioequivalence study based on LC-MS/MS method for the simultaneous determination of ALLP and its active metabolite, OXP in human plasma and urine, using 2,6-dichloropurine as the internal standard (IS). Simple and rapid liquid–liquid extraction was adopted, and ethyl acetate was used as the extraction agent. Ethyl acetate was used to extract analytes (0.5 ml aliquots of plasma or urine) and IS and separated on an Agilent Eclipse Plus C18 column using methanol and ammonium formate–formic acid buffer containing 5 mM ammonium formate and 0.1% formic acid (95 : 5, v/v) as the mobile phase (A) for ALLP or methanol: 5 mM ammonium formate aqueous solution (95 : 5, v/v) as the mobile phase (B) for OXP. ALLP was detected in positive ion mode, and the analysis time was about 7 min. The drug ALLP was linear from 0.05 to 5 g mL^−1^ in plasma and 0.5–30 g mL^−1^ in urine. The lower limit of quantification (LLOQ) was 0.05 g mL^−1^ in plasma and 0.5 g mL^−1^ in urine. The intra- and interday precision and relative errors of quality control (QC) samples were ≤11.1% for plasma and ≤8.7% for urine. OXP was detected in negative mode with an analysis time of about 4 min. The calibration curve was linear from 0.05 to 5 g mL^−1^ in plasma (LLOQ, 0.05 g mL^−1^) and from 1 to 50 g mL^−1^ in urine (LLOQ, 1 g mL^−1^). The intra- and interday precision and relative errors were ≤7.0% for plasma and ≤9.6% for urine. This method was then successfully applied to investigate the pharmacokinetics of ALLP and OXP in humans [41].

#### 3.5.2. GC-MS

Pechlivanis et al. have established GC-MS method to study the metabonomic investigation of the effects of physical exercise with ALLP administration in Wistar rats. Analysis was performed on an Agilent 7890A GC coupled to a 5975C Inert XL EI/CI MSD with Triple–Axis Detector MS and a CTCCH 4222 autosampler, using helium as a carrier gas at a flow of 3 mL min^−1^. Methoxyamine hydrochloride (MeOX) and N-methyl-N-(trimethylsilyl)trifluoroacetamide (MSTFA) are two techniques that the authors used for the derivatization of metabolite in this study. The researchers have studied the influence of allopurinol on the metabolic profile of blood plasma of rats that had undergone exhaustive swimming, and it was investigated by GC-MS. The programmed temperature vaporizing injector was ramped by 720°C min^−1^ from 100 to 270°C, where it was kept for 1 min, and then from 270 to 350°C, where it was kept for 5 min. The GC oven was ramped by 5°C min^−1^ from 70°C (2 min, initial time) to 200°C (1 min, stable) and from 200 to 320°C (5 min, stable). The total run time was 45 min per sample. Separation of the study groups as per the exercise was mainly due to lactic acid, pyruvic acid, 2-hydroxybutyric acid, uracil, oxalic acid, pyroglutamic acid, and stearic acid (*p* < 0.05). Lactic and pyruvic acids, indicating increased carbohydrate breakdown, and also 2-hydroxybutyric and pyroglutamic acids, stating increased glutathione synthesis in response to oxidative stress, were among the major differentiators of exercise from the resting state. Inosine, hypoxanthine, xanthine, xanthosine, and uric acid, indicating xanthine oxidase inhibition, as well as methionine, proline, and leucine, indicating increased protein synthesis, were among the major differentiators of ALLP administration from placebo. Results reveal that, despite having effects on metabolism and the redox status, ALLP does not seem to modulate the metabolic responses to exercise. Electron ionization source 50–800 m/z was used for the detection and quantification of the analyte by mass spectrometry [42].

#### 3.5.3. UPLC-MS/MS

Iqbal et al. have developed ultraperformance hydrophilic interaction liquid chromatography coupled with tandem mass spectrometry for simultaneous determination of ALLP, OXP, and LSD in rat plasma. Liquid–liquid extraction using ethyl acetate as extracting agent was used for sample extraction procedure. Acquity UPLC HILIC column (100 mm × 2.1, 1.7 *μ*m) was used as stationary phase in the presence of internal standard (5-fluorouracil). The mobile phase, consisting of acetonitrile, water, and formic acid (95 : 5:0.1, v/v/v), was eluted at 0.3 mL min^−1^ flow rate having total chromatographic run time of 3 min per sample. The analytes were detected on Acquity Triple Quadrupole Mass Spectrometer equipped with Z-Spray Electrospray Ionization (ESI). The calibration curve was found to be linear between 22 and 8000 ng mL^−1^ for ALLP, 33–12000 ng mL^−1^ for OXP, and 25–9000 ng mL^−1^ for LSD, respectively. The intra and interday precision (% RSD) for ALLP, OXP, and LES were found to be ≤10.54, ≤13.98, and ≤14.84%, respectively, whereas the intra and interday accuracy were within the range of 90.40–111.21%, 95.16–111.13%, and 91.92–108.30%, respectively. The mean absolute recovery of ALLP, OXP, and LES was found to be 79.42, 66.89, and 55.95%, respectively [43].

### 3.6. Miscellaneous Methods

#### 3.6.1. Electrophoresis

Pérez-Ruiz et al. have developed capillary zone electrophoresis method with UV absorbance detection for ALLP and its metabolite OXP, using 15 mM buffer adjusted at pH 8.8 as electrophoretic electrolyte equipped with P/ACE 5000 coupled to a diode array detector as electrophoresis instrument. The voltage and temperature during the analysis were 15 kV and 30°C, respectively. In this method, the researchers have optimized the electrophoresis conditions for ALLP and OXP, and they have also studied the effects of pH, type of buffer and its concentration, voltage effect on mobility, resolution, sensitivity, and speed. They found that the electrophoretic mobility of ALLP and OXP decreased continuously with the increase in the pH of buffer. The LOD obtained was 0.08 *μ*g mL^−1^ and 0.12 *μ*g mL^− 1^ for ALLP and OXP, respectively. The LOQ was 0.58 and 0.67 *μ*g mL^−1^ for ALLP and OXP, respectively. The drug was linear over the concentration of 0.68–96 *μ*g mL^−1^ for ALLP and 0.77–154 *μ*g mL^−1^ for OXP. The usefulness of this method is demonstrated by the excellent results obtained in the determination of ALLP and OXP in human serum and ALLP in different pharmaceutical formulations [44].

Sun et al. have established another electrophoresis method for end-column amperometric detection based on the responses of ALLP and OXP at carbon fiber electrode. The optimal conditions for the separation include buffer composed of 15 mM Na_2_HPO_4_/NaH_2_PO_4_ at a pH 9.55, electrokinetic injection 7s at 5 kV, separation voltage at 15 kV, and detection potential at 1.20 V. Effects of pH, separation voltage, buffer concentration, injection voltage, and time were analyzed. Validation of analytical parameters was as per ICH guidelines. LOD was found to be 1 × 10^−8^ mol L^−1^ for ALLP and OXP, and drug was found to be linear over the concentration of 2 × 10^−7^ to 1 × 10^−4^ mol L^−1^ and 1 × 10^−7^ to 1 × 10^−4^ for ALLP and OXP, respectively. The applicability of the developed method was established by spiking diluted urine sample (V_urine_ : V_buffer_ = 1 : 8) with ALLP and OXP, which produced favorable result [45].

Eman has developed electrophoresis method for the separation and determination of LSD and ALLP in their bulk, combined dosage form and in the presence of their degradation products under different stress conditions. Additionally, this is the first article to study the forced degradation and degradation kinetics to investigate the stability and half-life of the mixture at room temperature. Separation was carried out by using fused silica capillary (55 cm × 50 *μ*m id) using 50 mM borate buffer adjusted to pH 10 with 0.5 M NaOH. The drugs were found to be linear over the concentration of 5–50 and 10–100 *μ*g mL^−1^ for LSD and ALLP, respectively. Force degradation study of drugs showed that LSD was more liable for acidic and alkaline degradation and that ALLP degraded more by oxidation. The applicability of the developed method was established on tablet formulation which showed good result [46].

#### 3.6.2. Polarographic Method

Tommaso and Cataldi have developed anodic polarographic detection for ALLP at a mercury electrode using 0.05 M borate buffer. The flow injection-anodic polarographic detection method provided an accurate and sensitive drug determination with a detection limit of 1.8 *μ*m and a relative standard deviation of 3.1% at the 28 *μ*m level. The applicability of the developed method was established in commercially available ALLP tablets, and the recovery was found to be 99.6% with an RSD of 1.1% [47].

#### 3.6.3. Electrochemical Sensor Method

Ladmakhi et al. have developed a sensitive, selective, and precise electrochemical sensor based on Fe_3_O_4_@GO/OMC hybrid film on a carbon paste electrode for the determination of ALLP. The results showed sphere shape Fe_3_O_4_ nanoparticles with a diameter in the range 17–22 nm on composite. Modification of carbon paste electrode (CPE) with Fe_3_O_4_@GO/OMC (Fe_3_O_4_@GO/OMC-CPE) allowed the ultrasensitive and selective detection of ALO at oxidation potential of 1.05 V with linear range of 0.05–7 *μ*mol L^−1^, limit of detection of 47 nmol L^−1^, and sensitivity of 708 *μ*A mmol^−1^ L. The proposed sensor offers a simple and fast way for ALLP sensing in clinical samples within short analysis time, making the concept of interest. 500 *μ*L of human serum plasma or 1 mg of tablet was taken for analysis. The determinations of samples were also carried out by UV-visible spectroscopy, and characterizations were done by Raman and X-ray diffraction. Raman analysis of GO and Fe_3_O_4_@GO revealed the introduction of defects in the graphene framework after decoration of GO by Fe_3_O_4_ nanoparticles. The D and the G bands of GO appeared at 1323 and 1577 cm^−1^ with the intensity ratio of D and G bands (ID/IG) of 1.14. X-ray diffractogram revealed that the diffraction peaks at 32.1°, 35.8°, 37.4°, 43.8°, 52.4°, 57.3°, and 63.5 of Fe_3_O_4_@GO composite are in good agreement with the face-centered cubic spinel structure of Fe_3_O_4_ nanoparticles [48].

#### 3.6.4. Colorimetric Paper-Based Analytical Method

Pratiwi et al. have established a novel design and optimization of colorimetric paper-based analytical device for rapid detection of ALLP in herbal medicine. In this work, nine colorimetric reagents were screened to find the best colorimetric reagent for ALLP detection. These nine colorimetric reagents were chosen based on the reaction between the functional group on ALLP and a general reagent that was used as a colorimetric reagent. They are Dragendorff reagent, ferric chloride, Folin–Ciocalteu reagent, sodium nitroprusside, p-DAB reagent, Schiff reagent, potassium chlorate, Tollens reagent, and sodium nitrite. To calculate the levels of ALLP in herbal sample, standard curve of ALLP with concentrations of 6–16 *μ*g mL^−1^ was made and measured at 252 nm wavelength, and the concentration of ALLP in sample was determined (7.44 *μ*g mL^−1^). The developed paper-based analytical device was successful in detecting ALLP in herbal medicine sample which also agrees with TLC and spectrophotometry data [49].

#### 3.6.5. Near-Infrared Spectroscopy

Smetisko and Miljanic have developed a NIR spectroscopic method for assessment of drug dissolution from ALLP immediate release tablets. Thirty-three different batches of ALLP immediate release tablets containing constant amount of the active ingredient, but varying in excipient content and physical properties, were introduced in a PLS calibration model. The dissolution values were measured by UV-Vis method, and the data extracted from the NIR spectra, values of correlation coefficient, bias, slope, residual prediction determination, and root mean square error of prediction (0.9632, 0.328%, 1.001, 3.58, 3.75%) were evaluated. The obtained values revealed that the NIR diffuse reflectance spectroscopy can serve as a faster and simpler substitute to the conventional dissolution procedure, even for the tablets with a very fast dissolution rate (>85% in 15 minutes). The spectral region from 7158.9 cm^−1^ to 5484.9 cm^−1^ was chosen for the final calibration model, and the broad bands at 6872, 6493, and 6265 cm^−1^ resulted from lactose monohydrate. The distinctive ALLP bands were observed at 6092 and 6060 cm^−1^ and were allocated to the first overtone of the =CH stretching. Despite the large differences in the samples included in the calibration model, the developed method is not complicated and shows acceptable accuracy [50].

## 4. Conclusion

The present review provides a summary of various analytical methods reported in the literature for the determination of ALLP in bulk, pharmaceutical formulations and also in various biological matrices like blood plasma and urine. Analytical methods consisting of chromatography, spectroscopy, hyphenated techniques, and electrochemical methods were employed for determination of ALLP in bulk, pharmaceutical dosage forms and biological matrix.

The primary objective of the compilation of review is to collect maximum information available on analytical methods of ALLP and study it in detail. From this survey, it is revealed that a handful of analytical methods are obtainable on HPLC and UV-visible spectrophotometry and very few articles are available based on hyphenated methods and electrochemical methods.

The reported data for analysis of ALLP revealed that HPLC with UV detection is the most frequent technique employed for the determination of ALLP in pharmaceutical matrix. For analysis of ALLP in biological matrices like blood plasma, urine HPLC with UV detection is appropriate since this strategy gives precise outcomes and minimal effort. Furthermore, employing MS techniques in LC offered unique selectivity and sensitivity as well as a choice of method for analysis of ALLP and its metabolites in biological samples. Hyphenated techniques such as GC-MS, LC-MS, LC-MS/MS, and UPLC-MS/MS methods are also reported for quantification of ALLP in plasma and other biological fluids.

## Figures and Tables

**Table 1:** UV-visible and spectrofluorimetric methods for the determination of allopurinol.

S. no.	Drugs	Pharmaceutical matrix	Wavelength (nm)	Linearity (*μ*g·mL^−1^)	Assay (%)	Accuracy of the study (%)	Ref.
*UV-visible spectrophotometric methods*							
1.	ALLP	Tablet	580	2–10	98.2–102.5	100	[16]
2.	ALLP and LA	Tablet	250 and 310–390	10–50	101.2 and 100.69	101.18–101.61 and 100.81–101.84	[17]
3.	ALLP	Tablet	450 and 515	10–70	101.1 and 101.4	98.40–100.7 and 98.20–100.4	[18]
4.	ALLP and UA	Urine	284 and 293	0.2–1.2 mg/100 ml	99.25 and 98,97	100.14 and 100.23	[19]
5.	ALLP and FC	Tablet	281 and 275	0.25–3.5	99.01–102.24 and 99.88–101.89	99.75 and 99.84	[20]
6.	ALLP and LSD	Tablet	252–228, 240, and 290	1–16 *μ*g mL^–1^ and 3–45 *μ*g mL^–1^	99.98–100.23 and 100.05	99.77–100.22 and 100.26	[21]

*Spectrofluorimetry*							
7.	CM, TB, CB, and ALLP	Tablet	535/365	1–6 for CM and CB, 0.75–6 for TB, and 4–16 for ALLP	—	99.75–101.04	[23]
8.	LSD and ALLP	Tablet and human plasma	288/343 465/535	0.25–4.0 *μ*g mL^–1^ for LSD and 0.2–20 *μ*g mL^–1^ for ALLP	100.41 and 99.28	99.55 and 100.18	[24]

**Table 2:** HPLC/UPLC methods for the determination of allopurinol.

S. no.	Drugs	Pharmaceutical or biological matrix	Stationary phase	Chromatographic conditions	Linearity (*μ*g/ml)	Wavelength (nm)	Ref.
1	ALLP and LSD	Tablet	Zorbax C18 (4.6 × 150 mm × 5 *μ*m), injection volume = 10 *μ*L	Methanol: Phosphate buffer pH 3.9 (55 : 45v/v), flow rate = 1.0 mL min^−1^	100–500 *μ*g mL^−1^ for ALLP and 1–5 *μ*g mL^−1^ for LSD	255	[25]

2	ALLP and LSD	Tablet	Inertsil ODS (4.6 × 250 mm, 5 mm), injection volume = 20 *μ*L	Trifluoroacetic acid: methanol (40 : 60v/v), flow rate = 1.0 mL min^−1^	30–150 *μ*g mL^−1^ for ALLP and 20–100 *μ*g mL^−1^ for LSD	255	[26]

3	ALLP and OXP	Human plasma	Reversed-phase LiChrospher 100 RP-18 column (5 m; 250 × 4 mm; Merck, Darmstadt, Germany), flow rate = 1.0 mL min^−1^, injection volume = 40 *μ*L	0.02 M sodium acetate adjusted with acetic acid 30% to pH 4.5, flow rate = 1.0 mL min^−1^	0.5–10 mg L^−1^ for ALLP and 1.0–40 mg L^−1^ for OXP	254	[27]

4	ALLP and LPA	Tablet	Reversed-phase, Enable C18 G (250X4.6X5), injection volume = 20 *μ*L	Acetonitrile: 0.02 M ammonium acetate buffer adjusted to pH 4.6 in the proportion of 50 : 50 v/v, flow rate = 0.8 mL min^−1^	50–175 *μ*g mL^−1^ for both drugs	210	[28]

5	ALLP	Tablet	C8 (250 × 4.6 mm, 5 mm), injection volume = 10 *μ*L	70% buffer solution (monobasic ammonium phosphate 0.05 M with 30% acetonitrile and methanol 1 : 1), flow rate = 1 mL min^−1^	10–50 *μ*g mL^−1^	250	[29]

6	ALLP and KTZ	Capsule	InertSustain® C18 column (4.6 × 100 mm) *x* 3 *μ*m, injection volume = 20 *μ*L	Acetonitrile: water (52 : 48 v/v) with pH adjusted to 3.0, flow rate of 0.45 mL min^−1^	2.0 to 16.0 *μ*g mL^−1^ for ALLP and 1.0 to 15.0 *μ*g mL^−1^ for KTZ	ALLP at 250, KTZ at 225	[30]

7	ALLP, OXP, and UA	Urine and serum	RP (PerkinElmer RP-8, 10 pm, 250 × 4.6 mm), injection volume = 20 *μ*L	0.025 M phosphate buffer pH 6.1 with 6–8% of methanol, flow rate of 1.5 mL min^−1^	2–2000 ng in all matrices	+1.2 V vs. SCE for voltammetry, +0.24 V vs. Ag/AgCl for polarography,	[31]

8	ALLP and OXP	Human plasma and urine	Stainless steel tubes 6.35 mm (1/4 in.) OD, 4.5 mm ID, 30 mm and 70 mm long, packed with Aminex A-27 (12–15 *μ*m) anion-exchange resin	Ammonium acetate pH 8.7 at a flow rate of 1 mL min^−1^	For ALLP, 0.068–1.36 *μ*g mL^−1^, 0.68–136 *μ*g mL^−1^; for OXP, 0.076–15.2 *μ*g mL^−1^, 15.2–304 *μ*g mL^−1^ in plasma and urine, respectively	254	[32]

9	ALLP and OXP	Human serum	C18 (particle size 10 *μ*m, Waters co., Milford, MA, USA)	2% (v/v) acetonitrile solution-100 mm potassium phosphate (pH 4·0) and 0·5 mm tetra-n‐butylammonium hydrogen sulphate	For ALLP, 0·5–5·0 *μ*g mL^−1^; for OXP, 0·4–20 *μ*g mL^−1^	260	[33]

10	HPX XN, ALLP, OXP, and UA	Urine	HPLC: C18 Altex Model 310 (Altex Instruments, Berkeley, Calif.), GC: SE-30 (4% on 100/120 mesh SUPELCOPORT) glass column, 0.2 × 180 cm	HPLC: acetonitrile-buffer (1 : 1), GC: nitrogen as carrier gas at 20 mL min^−1^	40 to 500 *μ*g mL^−1^ for all samples	254 and 280	[34]

11	ALLP, OXP, HPX, XN	Urine and human plasma	ODS column (15 cm × 4.6 mm ID) and the precolumn (5 cm × 4.6 mm ID)	0.02 M potassium dihydrogen phosphate, pH adjusted to 3.65 with orthophosphoric acid	0.15 to 20 mg L^−1^ for ALLP and OXP; 0.50 to 50 *μ*mol L^−1^ for HPX and XN	254	[35]

12	ALLP, OXP	Human plasma	100 mm × 4.6 m ID and slurry packed with 5 *μ*m Hypersil ODS	20 mM disodium hydrogen phosphate dehydrate, pH 2.0 with orthophosphoric acid	0·05–5·0 *μ*g mL^−1^ for both drugs	254	[36]

## Data Availability

The data used to support the findings of this study are included within the article.
